# Significant association between the endothelial lipase gene 584C/T polymorphism and coronary artery disease risk

**DOI:** 10.1042/BSR20200027

**Published:** 2020-09-16

**Authors:** Yue-e Wu, Lan Ma, Hao Zhang, Xin-ran Chen, Xin-yi Xu, Ze-ping Hu

**Affiliations:** 1 Department of Electrocardiogram Diagnosis, The Second Affiliated Hospital, Anhui Medical University, Hefei, Anhui 230060, People’s Republic of China; 2 Department of Emergency, The Second Affiliated Hospital, Anhui Medical University, Hefei 230060, Anhui Province, People’s Republic of China; 3 Department of Cardiology, The First Affiliated Hospital, Anhui Medical University, Hefei 230022, Anhui Province, People’s Republic of China

**Keywords:** Coronary artery disease, Endothelial lipase gene, Meta-analysis, Polymorphism

## Abstract

Several studies have investigated a potential association between the endothelial lipase gene (*LIPG*) 584C/T polymorphism and susceptibility to coronary artery disease (CAD), but a uniform conclusion is yet to be reached. To better evaluate the true relationship between the *LIPG* 584C/T polymorphism and the risk of CAD, a meta-analysis of 14 case–control studies with 9731 subjects was performed. Relevant articles published through August 2020 were searched in the CNKI, PubMed, Embase and Web of Science databases. Thirteen articles, including 14 eligible case–control studies with 4025 cases and 5706 controls, were enrolled in the present meta-analysis. The Newcastle–Ottawa Scale (NOS) scores of the case–control studies ranged from 6 to 8. The pooled results indicated that there is a significant association between the *LIPG* 584C/T polymorphism and CAD in the homozygote comparison model and the allelic comparison model. Subgroup analyses revealed that the *LIPG* 584C/T mutation significantly decreased the risk of CAD in the subgroups of African, CAD, hospital-based (HB), and polymerase chain reaction-restriction fragment length polymorphism (PCR-RFLP) populations in some genetic models. No publication bias was found in our meta-analysis, which certifies the robustness of the current meta-analysis. Trial sequential analysis (TSA) also confirmed the stability of our results. The results of our meta-analysis indicate that the *LIPG* 584C/T polymorphism plays a protective role in the incidence of CAD. More high-quality case–control studies on various ethnicities are needed to confirm our results.

## Introduction

Coronary artery disease (CAD), also called coronary heart disease (CHD), characterized by myocardial hypoxia and ischemia is triggered by coronary atherosclerosis [1]. It is a medical problem of human society and one of the leading causes of disability and deaths in every country [2,3]. The precise mechanism of the onset and development of CAD is still obscure. The pathogenesis of CAD involves many risk factors, including hereditary and environmental factors. Recently, increasing evidence has suggested that genetic factors may play an important role in its pathogenesis. Many genetic variants have been identified to be related to the risk of CAD by previous genetic association studies [4]. The single-nucleotide polymorphism (SNP) is one of the most common genetic variants. Some SNPs may be related to the occurrence and development of CAD, while others are not [5,6].

Endothelial lipase (EL), a new member of the triglyceride (TG) lipase family, was first discovered in 1999. EL consists of 483 amino acids, with a molecular weight of approximately 55 kDa and is mainly secreted from endothelial cells and other cells such as hepatocytes and macrophages [7]. Compared with lipoprotein lipase (LPL) and hepatic lipase (HL), which are also members of the TG lipase family, EL has higher phospholipase activity and lower TG lipase. A large number of studies have demonstrated that EL plays a key role in high-density lipoprotein (HDL) metabolism. As a regulator of plasma HDL-C levels, EL can modulate the levels of plasma HDL-C inversely [8]. Elevated plasma HDL levels are associated with a protective effect on CAD and low plasma HDL levels are associated with an elevated risk of CAD [9]. Recently, increasing evidence has indicated that EL plays a pivotal role in the pathogenesis of CAD in part by reducing plasma HDL levels.

The EL gene (*LIPG*), which encodes the EL protein and is located on chromosome 18q21.1, spans approximately 30 kb with ten exons and nine introns [10]. Several genetic polymorphisms have been confirmed in *LIPG*. Among these, the 584C/T gene variant (rs2000813) has been the most frequently investigated variant that can lead the amino acid threonine substitution for isoleucine at codons [11]. Several published studies have assessed the association between *LIPG* 584C/T polymorphism and the risk of CAD; however, the results are still inconclusive and contradictory [12–14]. To testify these results, Cai et al*.* [15] carried out a meta-analysis to explore the association between the *LIPG* 584C/T variation and CAD risk in 2014. Unfortunately, the result of their meta-analysis was still inconclusive. Thereafter, several new studies have investigated the correlation between the *LIPG* 584C/T polymorphism and CAD risk, but the results of these studies remain inconsistent. Therefore, to elucidate the precise correlation of the *LIPG* 584C/T polymorphism with CAD susceptibility, we performed this updated meta-analysis.

## Materials and methods

### Search strategy

A systematic literature network search without language limitations was performed in the CNKI, PubMed, Embase and Web of Science databases to acquire all eligible case–control studies published before 15 August 2020. The relevant search terms included the following: (endothelial lipase gene OR EL OR *LIPG*) AND (polymorphism OR mutation OR variation OR genotype) AND (cardiovascular disease OR coronary heart disease OR coronary artery disease OR angina pectoris OR acute coronary syndrome OR myocardial infarction). Furthermore, to acquire other relevant published articles, the reference lists of the studies and reviews, which were included from the search parameters illuminated above, were searched.

### Inclusion and exclusion criteria

CAD was defined as acute myocardial infarction (AMI), CHD, CAD, myocardial infarction (MI), acute coronary syndrome (ACS), cardiovascular disease. The included articles fulfilled the following criteria: (1) studies assessed the correlation between the *LIPG* 584C/T polymorphism and CAD risk; (2) studies were case–control or cohort; (3) articles reported in Chinese or English; and (4) studies provided precise data for genotype distribution estimation in both groups. Studies were excluded according to the following criteria: (1) not case–control and cohort studies, letters, case reports, reviews or meta-analyses; (2) deficient data; (3) deviating from Hardy–Weinberg equilibrium (HWE) in the control group; and (4) overlapping data or duplicated publications.

### Data extraction

Two researchers (Yue-e Wu and Lan Ma) independently extracted data from the qualified articles based on the inclusion criteria. The following information was extracted from the eligible studies: first author, year of publication, ethnicity and country of participants, type of diseases, genotyping method, sources of controls, number of cases and controls for the 584C/T genotypes of *LIPG* and the *P*-value of the HWE test of controls. When we encountered inconsistent evaluations, all researchers were consulted to acquire an agreement regarding the exclusion or inclusion of the study in the present article.

### Methodological quality assessment

The Newcastle–Ottawa Scale (NOS) was used to evaluate the quality of each included study [16]. For the selection and exposure categories, each study can be awarded a maximum of one star for each numbered item. A maximum of two stars can be given for the comparability categories. The NOS has a score range of 0–9, and a study with a score higher than 5 could be included in the present meta-analysis.

### Statistical analysis

The present meta-analysis was conducted based on the Preferred Reporting Items for Systematic Reviews and Meta-Analyses (PRISMA) statement [17]. Chi-square test was applied to assess the HWE in the control groups with significance set at *P* less than 0.05. Odds ratios (ORs) with 95% confidence intervals (CIs) were used to evaluate the strength of the relationship between the *LIPG* 584C/T polymorphism and the risk of CAD in five genetic models: heterozygote comparisons (*CT vs. CC*), homozygote comparisons (*TT vs. CC*), recessive model (*TT vs. CT* + *CC*), dominant model (*TT* + *CT vs. CC*), and allelic comparisons (*T vs. C*). Stratified analyses were also performed according to ethnicity (Asian, Caucasian, and African populations), types of diseases (CHD, ACS, and CAD), genotyping method (polymerase chain reaction-restriction fragment length polymorphism (PCR-RFLP), Taqman, and PCR), and sources of controls (hospital-based (HB) and population-based (PB)). Differences in terms of a Z-test were considered statistically significant if the *P*-value was less than 0.05. The Cochran’s Q statistical test and the *I^2^* test were used to measure the heterogeneity within the studies, and the significance was set at *P* less than 0.05 [18]. A fixed-effects model or random-effects model was used to calculate the pooled OR according to the heterogeneity [19]. Influence analysis was carried out to examine the effect of an individual study on the pooled OR by omitting a single study each time to assess the stability of the pooled results. Publication bias was examined by Begg’s funnel plot test and Egger’s test [20,21]. Trial sequential analysis (TSA) was carried out as described in our previous study [22]. STATA 12.0 software (version 12.0; STATA Corp. College Station, TX, U.S.A.) was used to conduct all statistical analyses.

## Results

### Characteristics of eligible studies

**Figure 1:**
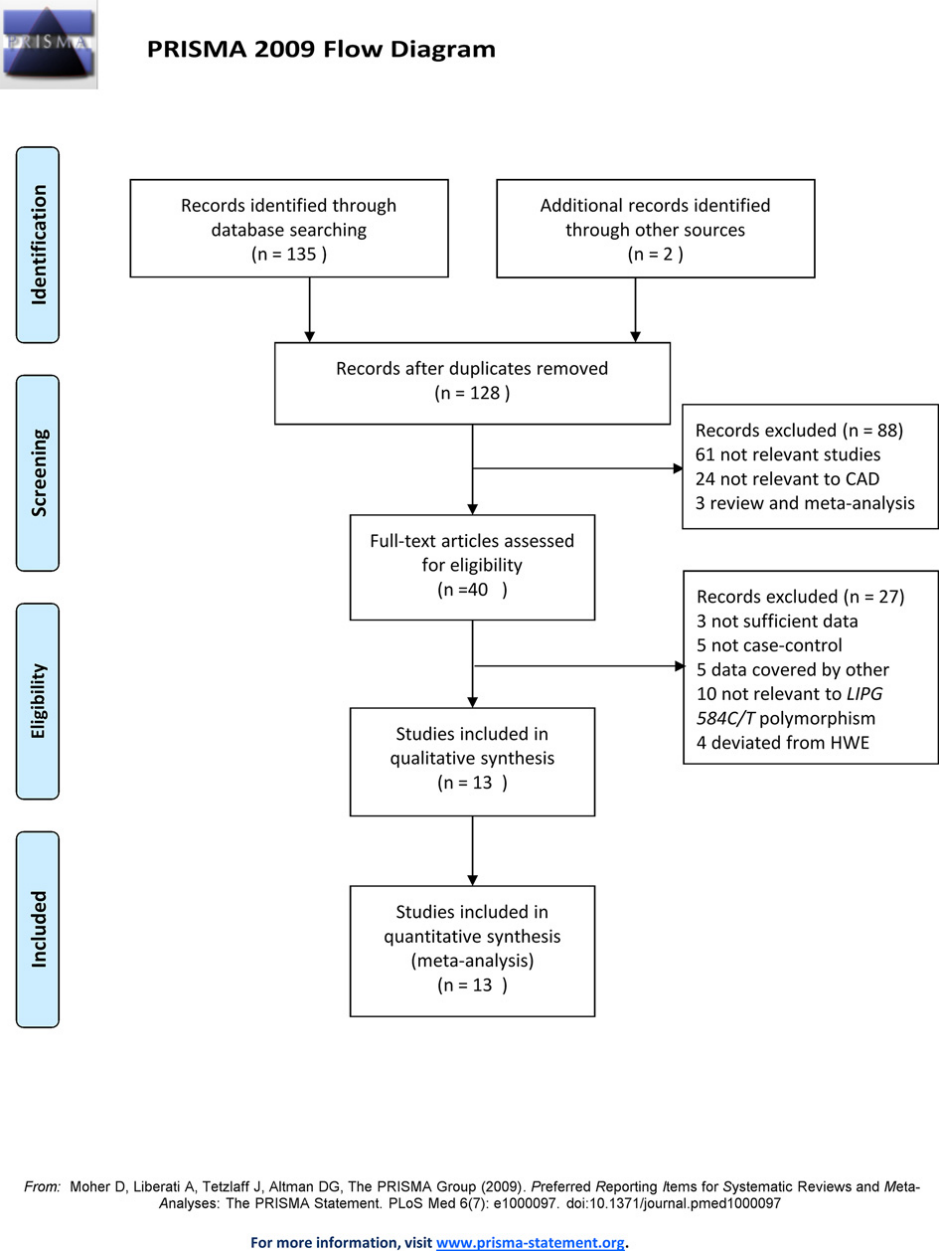
The flow diagram of the included and excluded studies

The process of screening eligible articles is shown in Figure 1. One hundred and thirty-seven potential articles were acquired after a systematic literature search. One hundred and twenty-four articles were removed after the comprehensive examining procedures according to the article titles, abstracts, and full-texts. Finally, the remaining 13 articles, including 14 eligible independent case–control studies with 4025 patients and 5706 controls, were identified and included in the meta-analysis based on the inclusion and exclusion criteria [9,12–14,23–31].

Table 1 displays the characteristics of all case–control studies included in our meta-analysis. All studies were reported in English except for two studies that were published in Chinese. Among the 14 studies, 7 studies were conducted in Caucasian populations, 6 in Asian populations, and only 1 in African populations. There were nine HB studies and five PB studies. Seven studies used PCR-RFLP as the genotyping method, four studies used TaqMan, and three studies used PCR. The genotype distributions of controls in all included studies were accordance with HWE. Table 2 shows the distribution of genotypes and allele frequencies of the *LIPG* 584C/T polymorphism in the cases and controls. Supplementary Table S1 indicated that the studies included in our study were reliable according to the NOS.

**Table 1:** Characteristics of eligible case–control studies included in this meta-analysis

First author	Year	Country	Ethnicity	Type of disease	Source of controls	Genotyping method	Number of cases	Number of controls	HWE (Control)	NOS score
Zhu et al. [12]	2007	China	Asian	CHD	HB	PCR-RFLP	242	196	0.063	7
Ji et al. [23]	2015	China	Asian	ACS	HB	PCR-RFLP	195	159	0.077	6
Jensen et al. [24]	2009	Denmark	Caucasian	ACS	PB	TaqMan	998	1643	0.888	8
Rimm et al. [25]	1992	America	Caucasian	CAD	PB	PCR	262	519	0.063	8
Colditz et al. [26]	1997	America	Caucasian	CAD	PB	PCR	241	477	0.220	8
Tjonneland et al.(1) [27]	2007	Denmark	Caucasian	CHD	PB	TaqMan	235	763	0.838	8
Tjonneland et al.(2) [27]	2007	Denmark	Caucasian	CHD	PB	TaqMan	763	880	0.997	8
Tang et al. [9]	2008	China	Asian	CAD	HB	PCR-RFLP	265	265	0.103	7
Xie et al. [13]	2015	China	Asian	CAD	HB	PCR	287	367	0.065	7
Solim et al. [28]	2018	Turkey	Caucasian	CAD	HB	TaqMan	74	73	0.545	7
Elnaggar et al. [29]	2019	Egypt	African	CAD	HB	PCR-RFLP	84	42	0.492	8
Dalan et al. [30]	2013	Turkey	Caucasian	CAD	HB	PCR-RFLP	104	76	0.286	7
Cai et al. [14]	2014	China	Asian	CAD	HB	PCR-RFLP	135	166	0.146	7
Toosi et al. [31]	2015	Iran	Asian	CAD	HB	PCR-RFLP	140	80	0.092	8

**Table 2:** *LIPG 584C/T* polymorphism genotype distribution and allele frequency in cases and controls

First author	Year	Genotype (*n*)								Allele frequency (*n*)				HWE (Control)
		Case				Control				Case		Control		
		Total	CC	CT	TT	Total	CC	CT	TT	C	T	C	T	
Zhu et al. [12]	2007	242	186	56	0	196	150	46	0	428	56	346	46	0.063
Ji et al. [23]	2015	195	112	76	7	159	81	71	7	300	90	233	85	0.077
Jensen et al. [24]	2009	998	509	406	83	1643	837	673	133	1424	572	2347	939	0.888
Rimm et al. [25]	1992	262	129	117	16	519	240	239	40	375	149	719	319	0.063
Colditz et al. [26]	1997	241	115	110	16	477	224	214	39	340	142	662	292	0.220
Tjonneland et al.(1) [27]	2007	235	116	102	17	763	391	312	60	334	136	1094	432	0.838
Tjonneland et al.(2) [27]	2007	763	393	304	66	880	446	361	73	1090	436	1253	507	0.997
Tang et al. [9]	2008	265	174	85	6	265	125	122	18	433	97	372	158	0.103
Xie et al. [13]	2015	287	160	116	11	367	216	139	12	436	138	571	163	0.065
Solim et al. [28]	2018	74	40	27	6	73	26	33	14	107	39	85	61	0.545
Elnaggar et al. [29]	2019	84	58	23	2	42	17	21	4	139	27	55	29	0.492
Dalan et al. [30]	2013	104	44	59	1	76	45	29	2	147	61	119	33	0.286
Cai et al. [14]	2014	135	84	45	6	166	97	64	5	213	57	258	74	0.146
Toosi et al. [31]	2015	140	67	70	3	80	28	46	6	204	76	102	58	0.092

### Test of heterogeneity

To assess the heterogeneity among the included studies, Q-test and *I^2^* statistics were applied. High heterogeneity was found across studies in all genetic models except for the recessive comparisons. Thus, random-effects analysis was applied to synthesize the data. Moreover, we explored the heterogeneity of all genetic models based on different ethnicities, type of diseases, genotyping method, and sources of controls. However, the significant heterogeneity could not be entirely explained by diverse ethnicities (Table 3).

**Table 3:** Meta-analysis results

Genetic model	Category	*n*	Model	OR (95% CI)	*P*	Heterogeneity		Begg’s test	Egger’s test
						*I^2^*	*P*	*P*	*P*
Homozygote (*TT vs. CC*)	Overall	14	R	0.740 [0.555; 0.987]	**0.041**	47.5%	0.029	0.059	0.121
	Asian	6	R	0.599 [0.275; 1.303]	0.196	61.2%	0.035	0.806	0.747
	Caucasian	7	F	0.920 [0.764; 1.110]	0.384	7.7%	0.370	0.072	0.115
	African	1	F	0.147 [0.025; 0.870]	**0.035**	-	-	-	-
	CHD	3	F	1.006 [0.741; 1.364]	0.972	0.0%	0.836	1.000	-
	ACS	2	F	1.002 [0.753; 1.333]	0.990	0.0%	0.542	1.000	-
	CAD	9	R	0.545 [0.336; 0.884]	**0.014**	49.6%	0.044	0.602	0.651
	HB	9	R	0.474 [0.255; 0.880]	**0.018**	50.6%	0.048	0.711	0.814
	PB	5	F	0.963 [0.796; 1.165]	0.696	0.0%	0.860	0.086	0.080
	PCR-RFLP	7	F	0.420 [0.253; 0.698]	**0.001**	39.1%	0.145	1.000	0.709
	Taqman	4	F	0.963 [0.783; 1.186]	0.725	44.4%	0.145	0.089	0.151
	PCR	3	F	0.850 [0.577; 1.252]	0.411	0.0%	0.613	0.296	0.077
Heterozygote (*CT vs. CC*)	Overall	14	R	0.883 [0.765; 1.031]	0.115	61.1%	0.001	0.063	0.441
	Asian	6	R	0.786 [0.599; 1.031]	0.082	59.5%	0.030	0.707	0.856
	Caucasian	7	F	0.994 [0.898; 1.101]	0.910	37.2%	0.145	1.000	0.790
	African	1	F	0.321 [0.144; 0.715]	**0.005**	-	-	-	-
	CHD	3	F	0.997 [0.850; 1.168]	0.966	0.0%	0.747	1.000	0.707
	ACS	2	F	0.961 [0.824; 0.715]	0.614	9.5%	0.293	1.000	-
	CAD	9	R	0.804 [0.605; 1.121]	0.131	72.6%	0.000	0.251	0.675
	HB	9	R	0.779 [0.574; 1.056]	0.108	71.5%	0.000	0.466	0.848
	PB	5	F	0.986 [0.888; 1.094]	0.788	0.0%	0.929	1.000	0.725
	PCR-RFLP	7	R	0.760 [0.529; 1.092]	0.138	72.5%	0.001	0.764	0.541
	Taqman	4	F	0.978 [0.871; 1.099]	0.712	14.4%	0.320	0.734	0.444
	PCR	3	F	1.006 [0.838; 1.208]	0.947	0.0%	0.643	1.000	0.421
Dominant (*TT* + *CT vs. CC*)	Overall	14	R	0.856 [0.725; 1.010]	0.066	68.3%	0.000	0.051	0.361
	Asian	6	R	0.772 [0.570; 1.044]	0.093	68.6%	0.007	0.707	0.898
	Caucasian	7	F	0.984 [0.893;1.085]	0.752	46.7%	0.081	0.764	0.996
	African	1	F	0.293 [0.135; 0.636]	**0.002**	-	-		
	CHD	3	F	0.998 [0.857; 1.162]	0.979	0.0%	0.830	1.000	0.739
	ACS	2	F	0.966 [0.834; 1.120]	0.649	22.0%	0.258	1.000	-
	CAD	9	R	0.764 [0.566; 1.032]	0.079	77.1%	0.000	0.348	0.649
	HB	9	R	0.745 [0.538; 1.031]	0.076	76.4%	0.000	0.466	0.802
	PB	5	F	0.983 [0.889; 1.086]	0.730	0.0%	0.923	0.806	0.922
	PCR-RFLP	7	R	0.734 [0.503; 1.072]	0.109	75.9%	0.000	0.764	0.529
	Taqman	4	F	0.977 [0.874; 1.091]	0.677	46.0%	0.136	0.734	0.326
	PCR	3	F	0.988 [0.828; 1.180]	0.898	0.0%	0.527	0.296	0.346
Recessive (*TT vs. CT + CC*)	Overall	14	F	0.881 [0.745; 1.041]	0.136	26.0%	0.182	0.100	0.103
	Asian	6	F	0.682 [0.435; 1.068]	0.094	47.1%	0.109	0.806	0.781
	Caucasian	7	F	0.932 [0.778; 1.117]	0.448	0.0%	0.529	**0.016**	**0.031**
	African	1	F	0.235 [0.041; 1.337]	0.103	-	-	-	-
	CHD	3	F	1.007 [0.750; 1.352]	0.962	0.0%	0.686	1.000	-
	ACS	2	F	1.013 [0.768; 1.336]	0.925	0.0%	0.668	1.000	-
	CAD	9	F	0.658 [0.487; 0.889]	**0.006**	26.7%	0.207	0.602	0.659
	HB	9	F	0.578 [0.390; 0.856]	0.006	30.6%	0.184	0.902	0.726
	PB	5	F	0.969 [0.806; 1.165]	0.136	0.0%	0.860	0.086	**0.035**
	PCR-RFLP	7	F	0.499 [0.302; 0.825]	**0.007**	21.3%	0.273	0.707	0.881
	Taqman	4	F	0.976 [0.798; 1.193]	0.814	18.7%	0.297	0.089	0.104
	PCR	3	F	0.855 [0.586; 1.246]	0.414	0.0%	0.700	0.296	**0.028**
Allelic (*T vs. C*)	Overall	14	R	0.869 [0.761; 0.992]	**0.037**	68.5%	0.000	0.080	0.288
	Asian	6	R	0.813 [0.630; 1.048]	0.110	69.7%	0.006	1.000	0.912
	Caucasian	7	F	0.978 [0.907; 1.055]	0.571	44.2%	0.096	0.174	0.484
	African	1	F	0.368 [0.200; 0.678]	**0.001**	-	-	-	-
	CHD	3	F	1.000 [0.886; 1.128]	0.999	0.0%	0.953	1.000	0.871
	ACS	2	F	0.981 [0.874; 1.102]	0.750	13.5%	0.282	1.000	-
	CAD	9	R	0.785 [0.622; 0.992]	**0.043**	76.3%	0.000	0.348	0.620
	HB	9	R	0.772 [0.595; 1.001]	0.051	75.3%	0.000	0.466	0.830
	PB	5	F	0.984 [0.910; 1.064]	0.682	0.0%	0.909	0.462	0.471
	PCR-RFLP	7	R	0.765 [0.571; 1.026]	0.074	73.0%	0.001	0.548	0.434
	Taqman	4	F	0.981 [0.900; 1.070]	0.669	58.0%	0.066	0.308	0.231
	PCR	3	F	0.971 [0.845; 1.117]	0.682	0.0%	0.467	0.296	**0.028**

Abbreviations: F, fixed-effects model; *n*, number of studies; R, random-effects model.

### Meta-analysis results

**Figure 2:**
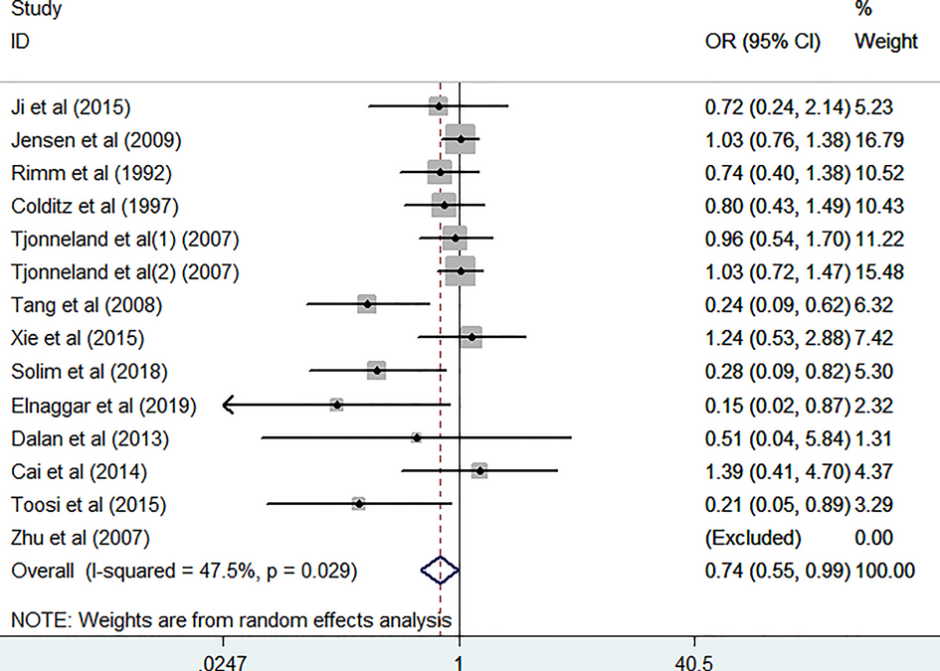
Forest plots of the *LIPG* 584C/T polymorphism and CAD risk (homozygote comparisons: *TT vs. CC*)

**Figure 3:**
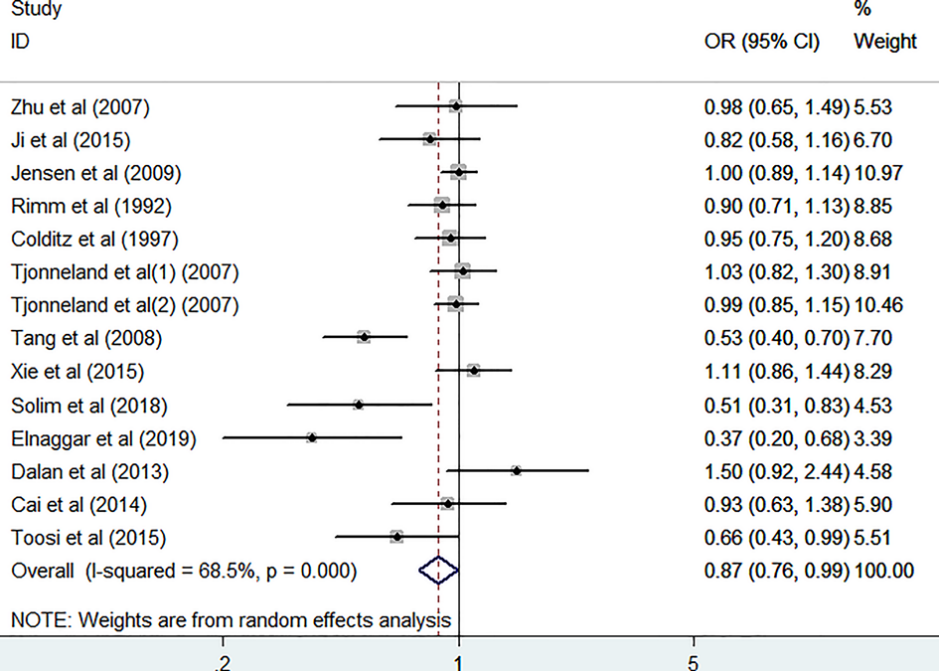
Forest plots of the *LIPG* 584C/T polymorphism and CAD risk (allelic comparisons: *T vs. C*)

The association between the *LIPG* 584C/T polymorphism and the risk of CAD was assessed. The integrated results indicated that the *LIPG* 584C/T variation was significantly associated with CAD risk in the homozygote comparison model (*TT vs. CC*: OR = 0.74, 95% CI = 0.56–0.99, *P*=0.041, *P*_heterogeneity_=0.029, Table 3 and Figure 2) and allelic comparison model (*T vs. C*: OR = 0.87, 95% CI = 0.76–0.99, *P*=0.037, *P*_heterogeneity_=0.000, Table 3 and Figure 3). There was no significant association with CAD risk in the other three models, including *CT vs. CC*: OR = 0.89, 95% CI = 0.77–1.03, *P*=0.115, *P*_heterogeneity_=0.001; *TT* + *CT vs. CC*: OR = 0.86, 95% CI = 0.73–1.01, *P*=0.066, *P*_heterogeneity_=0.000; and *TT vs. CT* + *CC*: OR = 0.88, 95% CI = 0.75–1.04, *P*=0.136, *P*_heterogeneity_=0.182 (Table 3).

In the subgroup analysis according to ethnicity, the results showed a decreased CAD risk in African populations under four genetic models (*TT vs. CC*: OR = 0.15, 95% CI = 0.03–0.87, *P*=0.035; *CT vs. CC*: OR = 0.32, 95% CI = 0.14–0.72, *P*=0.005; *TT* + *CT vs. CC*: OR = 0.29, 95% CI = 0.14–0.64, *P*=0.002; and *T vs. C*: OR = 0.37, 95% CI = 0.20–0.68, *P*=0.001). However, there was no significant association between the *LIPG* 584C/T polymorphism and the risk of CAD that was identified in Asian and Caucasian populations in all genetic models. In the subgroup analysis stratified by type of diseases, the results indicated that the *LIPG* 584C/T polymorphism was significantly associated with decreased CAD risk in the subgroup of CAD in three genetic models (*TT vs. CC*: OR = 0.55, 95% CI = 0.34–0.88, *P*=0.014; *TT vs. CT* + *CC*: OR = 0.66, 95% CI = 0.49–0.89, *P*=0.006; and *T vs. C*: OR = 0.79, 95% CI = 0.62–0.99, *P*=0.043), and the same results were shown in the HB group (*TT vs. CC*: OR = 0.48, 95% CI = 0.26–0.88, *P*=0.018) and PCR-RFLP (*TT vs. CC*: OR = 0.42, 95% CI = 0.25–0.70, *P*=0.001). Detailed results are shown in Table 3.

### Sensitivity analysis

**Figure 4:**
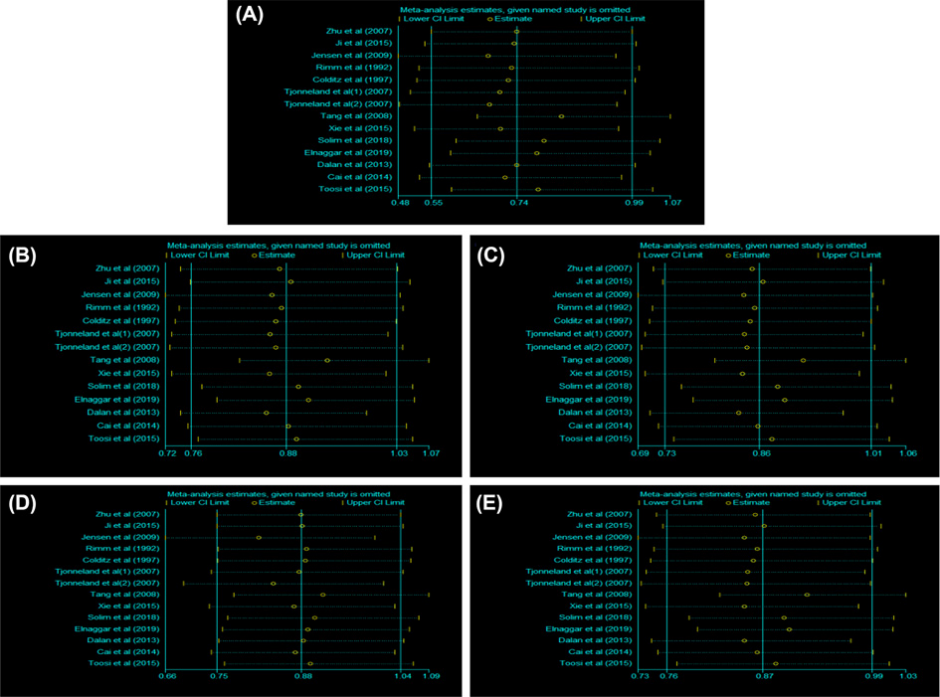
The results of sensitivity analysis between the *LIPG* 584C/T polymorphism and susceptibility to CAD (**A**) homozygous model; (**B**) heterozygous model; (**C**) dominant model; (**D**) recessive model; (**E**) allele model.

Sensitivity analysis was carried out to examine the influence of each eligible study on the pooled ORs by the sequential removal of each individual study form the analysis. The individual removal procedure affected the pooled ORs in the homozygote and allelic comparisons model, indicating the instability and unreliability of our findings. Sensitivity analyses of the other three genetic models showed similar results (Figure 4).

### Publication bias

**Figure 5:**
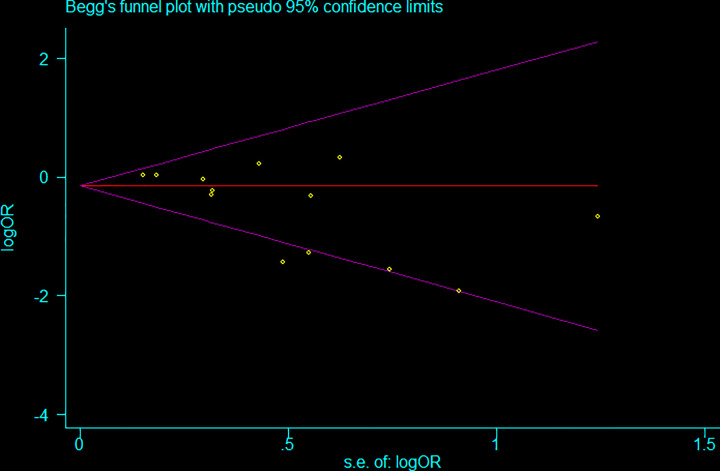
Funnel plot assessing evidence of publication bias (homozygote comparisons: *TT vs. CC*)

To assess the potential for publication bias among the studies on the the *LIPG* 584C/T polymorphism included in the present meta-analysis, Begg’s and Egger’s tests were performed for the five genetic models. No evidence of asymmetry was observed by the appearance of the shape in the Begg’s funnel plots, and neither Egger’s regression nor Begg’s rank correlation indicated publication bias among the studies in the five genetic models. The publication bias tests of subgroups were also performed, and no significant publication bias were found, except in few subgroups (Table 3 and Figure 5).

### TSA

**Figure 6:**
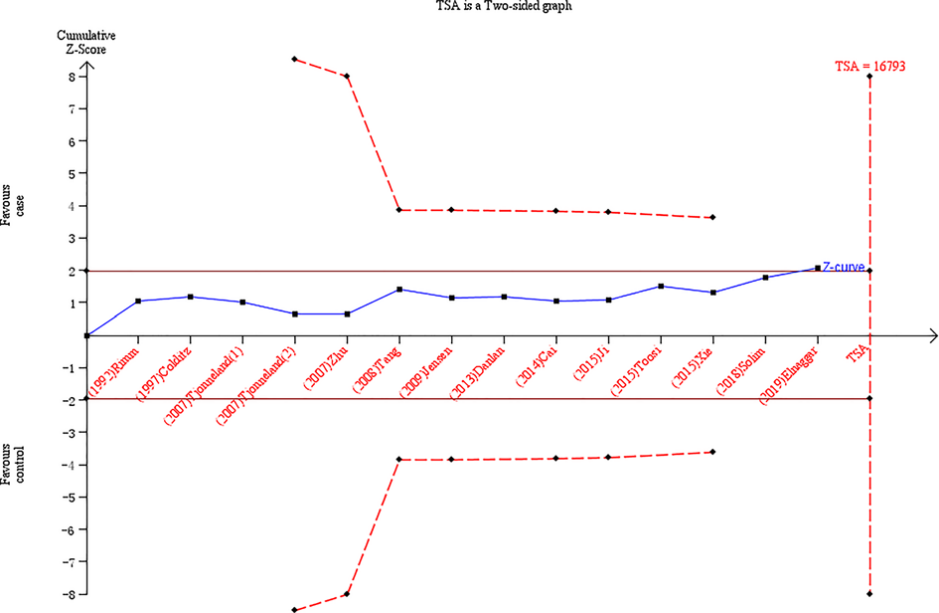
TSA for the *LIPG* 584C/T polymorphism and CAD risk in the homozygote comparisons

**Figure 7:**
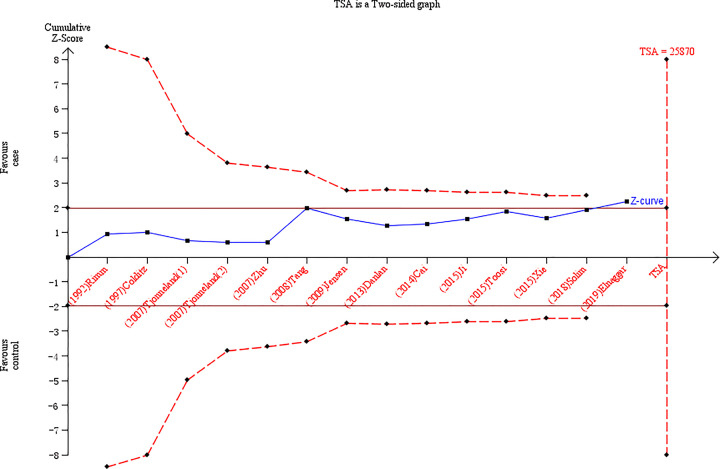
TSA for the *LIPG* 584C/T polymorphism and CAD risk in the allelic comparisons

The results of the TSA using the homozygote and allelic comparisons model are shown in Figures 6 and 7. The cumulative z-curve obviously crossed the traditional boundary, and it further confirmed the results of our meta-analysis that the *LIPG* 584C/T polymorphism was significantly associated with decreased CAD risk. Nevertheless, the cumulative z-curve had crossed the TSA monitoring boundary without success before reaching the required information size and demonstrated that the cumulative sample size is insufficient and the more eligible case–control studies are necessary.

## Discussion

The present meta-analysis indicated that the *LIPG* 584C/T variation was significantly associated with CAD risk in the homozygote comparison model (*TT vs. CC:* OR = 0.74, 95% CI = 0.56–0.99, *P*=0.041) and allelic comparison model (*T vs. C*: OR = 0.87, 95% CI = 0.76–0.99, *P*=0.037). According to the integrated analyses of 14 eligible case–control studies, our study revealed that the *LIPG* 584C/T polymorphism was significantly associated with a decreased risk of CAD, which suggested that the *LIPG* 584C/T polymorphism plays a protective role in the incidence of CAD in individuals and that the T allele may be a protective factor against CAD.

It has been proven that elevated plasma HDL levels are associated with a protective effect on CAD, and low plasma HDL levels are associated with an elevated risk of CAD [9]. The role of EL in the pathogenesis of CAD is regulated by its effect on the metabolism of HDL by its increasing clearance. Therefore, it is speculated that *LIPG* is closely related to the incidence of CAD by affecting the expression of EL. In recent years, more and more attention has been paid to genetic research on CAD, which is not surprising considering the impact of CAD on the health of people around the world [13]. To date, many molecular epidemiological studies have been performed to investigate the association between the *LIPG 58*4C/T variant and CAD risk. However, the results of these studies are inconsistent or even contradictory. The results found by Ji et al. [23], Zhu et al. [12], and Jensen et al. [24] revealed that there was no significant association between the *LIPG* 584C/T polymorphism and risk of CAD; in contrast, Tang et al. [9], Solim et al. [28], and Elnaggar et al. [29] found similar results as ours that the *LIPG* 584C/T polymorphism was significantly associated with a decreased risk of CAD. Cai et al. conducted a meta-analysis to investigate the true association between the *LIPG* 584C/T variant and CAD risk in 2014. However, their analysis included only 9 case–control studies, including 3036 cases and 4777 controls, and concluded that there was no significant association between the *LIPG* 584C/T polymorphism and the susceptibility to CAD. Their results were inconsistent with our study, but three case–control studies of their meta-analysis did not conform to HWE [32–34]. A latest meta-analysis of 13 published case–control studies was performed by Zhao et al. (2020) [35], and also significant results were found. However, in addition to also including three case–control studies that did not conform to HWE, some qualified studies were not included in their meta-analysis. Compared with their meta-analyses, the results of our meta-analysis, which included 14 case–control studies that all were in line with HWE, had more statistical power and reliability.

We noticed significant heterogeneities among the studies and carried out subgroup analyses according to ethnicity. According to previous studies, the *LIPG* 584C/T variant had different effects on CAD in different ethnicities. Even results from studies on the same ethnicity were inconsistent. For example, Tang et al. conducted a study including 530 Chinese subjects to assess the relationship between the common variant and CAD risk [9]. They demonstrated that the T allele could significantly reduce the CAD risk. The same results were found in studies conducted by Solim et al. [28] on the Turkish population, by Shimizu et al. [32] on the Japanese population, and by Toosi et al. [31] on the Iranian population. However, Jensen et al. [24] and Zhu et al. [12] showed no relationship between the *LIPG* 584C/T variant and CAD susceptibility in Caucasian and Asian populations. Our present study showed that the *LIPG* 584C/T variant be connected with a decreased CAD risk only in African populations. However, there was no significant association between the *LIPG* 584C/T polymorphism and the risk of CAD that was identified in Asian and Caucasian populations. This result was not convincing enough, because only one case–control study was included in the African subgroup; therefore, this result needs to be confirmed by more high-quality case–control studies in various ethnicities. When carrying out subgroup analysis by type of diseases, we found that the *LIPG* 584C/T polymorphism was significantly associated with a reduced risk in the CAD group, but we failed to find a significant risk association in other types. This result may be explained by the inherent heterogeneity of development in diverse diseases types.

Some inherent drawbacks of our meta-analysis should be illustrated when interpreting our results. First, only unadjusted estimates were applied to evaluate the strength of the relationship between the *LIPG* 584C/T variant and CAD risk. Due to a lack of raw data, such as exposing factors, life habits, gene–environment interactions, interactions between gene–gene and even diverse mutation loci in the same gene factors, a further exact adjustment analysis adjusting for confounding factors could not be carried out. Second, there is still high heterogeneity in our meta-analysis, although we used strict inclusion criteria, accurate data extraction, and rigorous data analysis to perform the present meta-analysis. The persuasiveness of the significant association between the *LIPG* 584C/T polymorphism and CAD susceptibility was affected. Third, the sample sizes of our study were still small, especially in subgroup analysis. Only one eligible study was included in the analysis of African populations. TSA identified that the cumulative sample size was insufficient. There were not enough eligible case–control studies, weakening the statistical power to detect the real correlation between the *LIPG* 584C/T polymorphism and susceptibility to CAD. Fourth, the result of our meta-analysis should be interpreted with caution and needs to be confirmed by more case–control studies, because the sensitivity analyses indicated that deletion of certain individual study had an impact on the reliability of our results. Fifth, although Begg’s and Egger’s tests proved that there was no publication bias, it may inevitably exist in our meta-analysis. Only published articles reported in English or Chinese were included in the present study. Some candidate studies may not be included because they were not published, discovered, or because they were not published in English or Chinese.

In conclusion, our meta-analysis suggested that the *LIPG* 584C/T polymorphism plays a protective role in the incidence of CAD in individuals. The T allele may be a protective factor against CAD. Because of the limitations mention above, more high-quality case–control studies in various ethnicities are needed to confirm our results.

## Supplementary Material

Supplementary Table S1

## Abbreviations

ACS: acute coronary syndrome
CAD: coronary artery disease
CHD: coronary heart disease
CI: confidence interval
CNKI: China National Knowledge Infrastructure
EL: endothelial lipase
HB: hospital-based
HDL: high-density lipoprotein
HDL-C: high-density lipoprotein cholesterol
HWE: Hardy–Weinberg equilibrium
LIPG: endothelial lipase gene
NOS: Newcastle–Ottawa Scale
OR: odds ratio
PB: population-based
PCR-RFLP: polymerase chain reaction-restriction fragment length polymorphism
SNP: single-nucleotide polymorphism
TG: triglyceride
TSA: trial sequential analysis

## References

[1] Willerson J.T. and Ridker P.M. (2004) Inflammation as a cardiovascular risk factor. Circulation 109, II2–II10 15173056 10.1161/01.CIR.0000129535.04194.38

[2] Moran A.E., Forouzanfar M.H., Roth G.A., Mensah G.A., Ezzati M., Flaxman A. et al. (2014) The global burden of ischemic heart disease in 1990 and 2010: the global burden of disease 2010 study. Circulation 129, 1493–1501 10.1161/CIRCULATIONAHA.113.004046 24573351 PMC4181601

[3] Global Burden of Disease Study 2013 Collaborators (2013) Global, regional, and national incidence, prevalence, and years lived with disability for 301 acute and chronic diseases and injuries in 188 countries, 1990-2013: a systematic analysis for the Global Burden of Disease Study 2013. Lancet 386, 743–800 10.1016/S0140-6736(15)60692-4 PMC4561509 26063472

[4] Sayols-Baixeras S., Lluís-Ganella C., Lucas G. and Elosua R. (2014) Pathogenesis of coronary artery disease: focus on genetic risk factors and identification of genetic variants. Appl. Clin. Genet. 7, 15–32 24520200 10.2147/TACG.S35301 PMC3920464

[5] Ji Y.N., Wang Q. and Zhan P. (2012) Intercellular adhesion molecule 1 gene K469E polymorphism is associated with coronary heart disease risk: a meta-analysis involving 12 studies. Mol. Biol. Rep. 39, 6043–6048 10.1007/s11033-011-1418-6 22203486

[6] Wang J., Zou L., Song Z., Lang X., Huang S., Lu F. et al. (2012) Meta-analysis of RAGE gene polymorphism and coronary heart disease risk. PLoS ONE 7, e50790 10.1371/journal.pone.0050790 23236395 PMC3516500

[7] Badellino K.O., Wolfe M.L., Reilly M.P. and Rader D.J. (2006) Endothelial lipaseconcentrations are increased in metabolic syndrome and associated with coronary atherosclerosis. PLoS Med. 3, e22 10.1371/journal.pmed.0030022 16354105 PMC1316064

[8] Yasuda T., Ishida T. and Rader D.J. (2010) Update on the role of endothelial lipase in high-density lipoprotein metabolism, reverse cholesterol transport, and atherosclerosis. J. Circ. 11, 2263–2270 10.1253/circj.CJ-10-0934 20962428

[9] Tang N.P., Wang L.S., Yang L., Zhou B., Gu H.J., Sun Q.M. et al. (2008) Protective effect of an endothelial lipase gene variant on coronary artery disease in a Chinese population. J. Lipid Res. 49, 369–375 10.1194/jlr.M700399-JLR200 17986713

[10] DeLemos A.S., Wolfe M.L., Long C.J., Sivapackianathan R. and Rader D.J. (2002) Identification of genetic variants in endothelial lipase in persons with elevated high-density lipoprotein cholesterol. Circulation 106, 1321–1326 10.1161/01.CIR.0000028423.07623.6A 12221047

[11] Paradis M.E., Couture P., Bosse Y., Despres J.P., Perusse L., Bouchard C. et al. (2003) The T111 I mutation in the EL gene modulates the impact of dietary fatonthe HDL profile in women. J. Lipid Res. 44, 1902–1908 10.1194/jlr.M300118-JLR200 12867537

[12] Zhu J.C., Jin G.D., Jin C.Y. and Xu G. (2009) Association of endothelial lipase gene Thr111Ile and Gly26Ser polymorphism with lipoprotein in patients with coronary heart disease. Chin. J. Pathophysiol. 23, 1684–1687

[13] Xie L., Sun Y., Tong Y., Liu Y. and Deng Y. (2015) Association of endothelial lipase gene-384A/C with coronary artery disease in Han Chinese people. BMJ Open 5, e007621, 29 10.1136/bmjopen-2015-007621 26124511 PMC4486941

[14] Cai G.J., He G.P., Huang Z.Y. and Qi C.P. (2014) Lack of association between a common polymorphism of the endothelial lipase gene and early-onset coronary artery disease in a Chinese Han population. Genet. Mol. Res. 20, 1059–1069 10.4238/2014.February.20.7 24634127

[15] Cai G., Huang Z., Zhang B., Weng W. and Shi G. (2014) The associations between endothelial lipase 584C/T polymorphism and HDL-C level and coronary heart disease susceptibility: a meta-analysis. Lipids Health Dis. 13, 85, 10.1186/1476-511X-13-85 24886585 PMC4041051

[16] Stang A. (2010) Critical evaluation of the Newcastle-Ottawa scale for the assessment of the quality of nonrandomized studies in meta-analyses. Eur. J. Epidemiol. 25, 603–605 10.1007/s10654-010-9491-z 20652370

[17] Moher D., Liberati A., Tetzlaff J. and Altman D.G. (2009) Preferred reporting items for systematic reviews and meta-analyses: the PRISMA statement. J. Clin. Epidemiol. 62, 1006–1012 10.1016/j.jclinepi.2009.06.005 19631508

[18] Higgins J.P., Thompson S.G., Deeks J.J. and Altman D.G. (2003) Measuring inconsistency in meta-analyses. BMJ 327, 557–560 10.1136/bmj.327.7414.557 12958120 PMC192859

[19] Higgins J.P. and Thompson S.G. (2002) Quantifying heterogeneity in a meta-analysis. Stat. Med. 21, 1539–1558 10.1002/sim.1186 12111919

[20] Begg C.B. and Mazumdar M. (1994) Operating characteristics of a rank correlation test for publication bias. Biometrics 50, 1088–1101 10.2307/2533446 7786990

[21] Egger M., Davey Smith G., Schneider M. and Minder C. (1997) Bias in meta-analysis detected by a simple, graphical test. BMJ 315, 629–634 10.1136/bmj.315.7109.629 9310563 PMC2127453

[22] Jiang Y., Li W., Lu J., Zhao X. and Li L. (2018) Association between PRKAA1 rs13361707T>C polymorphism and gastric cancer risk: evidence based on a meta-analysis. Medicine (Baltimore) 97, e0302 29620653 10.1097/MD.0000000000010302 PMC5902272

[23] Ji J.G., He G.P., Shi Y.W., Qian Y.C., Li W.H., Xue D.L. et al. (2015) The association between endothelial lipase gene 584C/T polymorphism and acute coronary syndrome in elderly patients in Changzhou. J. Nanjing Med. Univ. (Nat. Sci.) 35, 240–242

[24] Jensen M.K., Rimm E.B., Mukamal K.J., Edmondson A.C., Rader D.J., Vogel U. et al. (2009) The T111I variant in the endothelial lipase gene and risk of coronary heart disease in three independent populations. Eur. Heart J. 30, 1584–1589 10.1093/eurheartj/ehp145 19411665 PMC2733737

[25] Rimm E.B., Giovannucci E.L., Stampfer M.J., Colditz G.A., Litin L.B. and Willett W.C. (1992) Reproducibility and validity of an expanded self-administered semiquantitative food frequency questionnaire among male health professionals. Am. J. Epidemiol. 135, 1114–1126 10.1093/oxfordjournals.aje.a116211 1632423

[26] Colditz G.A., Manson J.E. and Hankinson S.E. (1997) The Nurses’ Health Study: 20-year contribution to the understanding of health among w omen. J. Womens Health 6, 49–62 10.1089/jwh.1997.6.49 9065374

[27] Tjønneland A., Olsen A., Boll K., Stripp C., Christensen J., Engholm G. et al. (2007) Study design, exposure variables, and socioeconomic determinants of participation in diet, cancer and health: a population-based prospective cohort study of 57,053 men and women in Denmark. Scand. J. Public Health 35, 432–441 10.1080/14034940601047986 17786808

[28] Solim L.A., Gencan I.A., Çelik B., Ataacar A., Koç U., Büyükören B. et al. (2018) Endothelial lipase gene polymorphism (584 C/T) in coronary artery patients among a Turkish population. In Vivo 32, 1105–1109 10.21873/invivo.11352 30150432 PMC6199614

[29] Elnaggar I.Z., Hussein S., Amin M.I. and Abdelaziz E.A. (2019) Association of 584C/T polymorphism in endothelial lipase gene with risk of coronary artery disease. J. Cell. Biochem. 120, 14414–14420 10.1002/jcb.28697 31020688

[30] Dalan A.B., Toptaş B., Buğra Z., Polat N., Yılmaz-Aydoğan H., Çimen A. et al. (2013) The effects of endothelial lipase gene (LIPG) variants on inflammation marker levels and atherosclerosis development. Mol. Biol. Rep. 40, 5143–5149 10.1007/s11033-013-2615-2 23673478

[31] Toosi S., Senemar S., Ahmadi Z. and Radmanesh S. (2015) Investigation of the association between 584C/T polymorphism of EL gene and risk of premature coronary artery disease in Fars province. J. Cardiovasc. Thorac. Res. 7, 118–121 10.15171/jcvtr.2015.25 26430500 PMC4586598

[32] Shimizu M., Kanazawa K., Hirata K., Ishida T., Hiraoka E., Matsuda Y. et al. (2007) Endothelial lipase gene polymorphism is associated with acute myocardial infarction, independently of high-density lipoprotein-cholesterol levels. Circ. J. 71, 842–846 10.1253/circj.71.842 17526978

[33] Cai G.J., He G.P. and Qi C.P. (2012) Association between endothelial lipase 584C/T gene polymorphism and acute coronary syndrome and lipids. Chin. Heart J. 24, 708–711 10.1007/s11033-012-1854-y 22723003

[34] Vergeer M., Cohn D.M., Boekholdt S.M., Sandhu M.S., Prins H.M., Ricketts S.L. et al. (2010) Lack of association between common genetic variation in endothelial lipase (LIPG) and the risk for CAD and DVT. Atherosclerosis 211, 558–564 10.1016/j.atherosclerosis.2010.04.004 20466371

[35] Zhao H., Zhao R., Hu S. and Rong J. (2020) Gene polymorphism associated with angiotensinogen (M235T), endotheliallipase (584C/T) and susceptibility to coronary artery disease: a meta-analysis. Biosci. Rep. 40, BSR20201414, 10.1042/BSR20201414 32667032 PMC7383830

