# Supplementary material for: Significant association between the endothelial lipase gene 584C/T polymorphism and coronary artery disease risk
Source: Biosci Rep. 2020 Sep 16;40(9):BSR20200027. doi: 10.1042/BSR20200027 (PMC7494996; doi:10.1042/BSR20200027)
Supplement: Supplementary Table S1 [file BSR-2020-0027_supp.pdf]

Supplemental Table 1 Methodological quality of the included studies according to the Newcastle-Ottawa Scale.

Study	Selection (score)				Comparability (score)	Exposure (score)			
	Adequate definition of patient case	Representativeness of patients cases	Selection of controls	Definition of control	Control for important factor or additional factor	Ascertainment of exposure	Same method of ascertainment for participants	Non-response Rate	Total Score
Zhu et al 2007 [12]	*	*	NA	*	**	*	*	NA	7
Cai et al 2014 [14]	*	*	NA	*	**	*	*	NA	7
Ji et al 2015 [23]	*	*	NA	*	*	*	*	NA	6
Jensen et al 2009 [24]	*	*	*	*	**	*	*	NA	8
Toosi et al 2015 [31]	*	*	NA	*	**	*	*	NA	7
Rimm et al 1992 [25]	*	*	*	*	**	*	*	NA	8
Colditz et al 1997 [26]	*	*	*	*	**	*	*	NA	8
Tjonneland et al 2007 [27]	*	*	*	*	**	*	*	NA	8
Tang et al 2008 [9]	*	*	NA	*	**	*	*	NA	7
Xie et al 2015 [13]	*	*	NA	*	**	*	*	NA	7
Solim et al 2018 [28]	*	*	NA	*	**	*	*	NA	7
Elnaggar et al 2019 [29]	*	*	NA	*	**	*	*	*	8
Dalan et al 2013 [30]	*	*	NA	*	**	*	*	NA	7
